# User-Centred Interaction Design for Enhancing Professional Well-Being in Healthcare Environments

**DOI:** 10.3390/healthcare14050637

**Published:** 2026-03-03

**Authors:** Maria Chiara Caschera, Tiziana Guzzo

**Affiliations:** Institute for Research on Population and Social Policies (IRPPS), National Research Council (CNR), Via Palestro 32, 00185 Rome, Italy; mariachiara.caschera@cnr.it

**Keywords:** user-centred design, user experience, Human–Computer Interaction (HCI), interaction design framework, inclusive design, user requirements

## Abstract

Background/Objectives: Adoption of user-centred design methods is essential in healthcare applications because it ensures that complex workflows are shaped around real users’ needs and behaviours, improving usability, accessibility, and sustainability. The use of user-centred design in healthcare applications still presents open challenges for identifying user requirements, including diverse stakeholder needs, limited user availability, complex interaction workflows, and organizational constraints. To address these challenges, this paper proposes a user-centred interaction design framework that systematically supports the identification and translation of user needs into actionable design requirements. Methods: The framework integrates user-centred design principles with generative tools, employing the Persona-and-Scenario method to transform user insights into actionable design requirements. By actively involving healthcare stakeholders, the framework ensures that both explicit and latent needs are captured. Results: The framework was implemented through two co-design events, which provided valuable feedback on data collection, visualization, interaction modalities, and privacy considerations. These insights were translated into functional, usability, and interface requirements for the Change Management Platform (CMP) for the KEEPCARING project. Conclusions: This framework introduces a structured, scenario-driven process that actively engages stakeholders in envisioning future states rather than merely refining existing systems. Its application demonstrates promising indications that it enhances requirement elicitation, promotes cross-stakeholder alignment, and yields higher-quality, contextually relevant design requirements.

## 1. Introduction

Healthcare applications, such as mobile health (mHealth) applications and clinical decision support systems, operate in environments characterized by high cognitive load, time pressure, and safety-critical decision-making. Poor usability in such systems has been linked to medical errors, clinician burnout, and reduced patient engagement [1,2].

In addition, research on detection methods for medical data analysis [3] and patient adoption of online medical advice through team-based consultation models [4] do not explicitly address how user requirements for healthcare platforms are collaboratively elicited, structured, and translated into design decisions.

To face this challenge, User-centred Design (UCD) methods [5] are increasingly recognized as fundamental for creating sustainable healthcare applications. Engaging users is a relevant source of insight into the context of use and should be utilized to explore potential design solutions. Furthermore, users’ active participation fosters a clearer understanding of their needs and expectations regarding the platforms. Traditional user-centred design methods (such as interviews, surveys, focus groups, and participatory workshops) have been widely adopted to capture user perspectives. These methods differ in their structure, stakeholder involvement, and methodological rigor, but they all aim to incorporate end-user perspectives to ensure relevance, usability, and broad adoption.

While these methods provide valuable insights, they also present several limitations, such as an incomplete articulation of latent needs, limited contextualization of real-world scenarios, and challenges in scalability and adaptability when applied to diverse user groups. Moreover, traditional methods tend to generate generalized feedback and primarily focus on evaluating existing ideas rather than fostering innovation. Participatory and collaborative workshops offer valuable insights; they often lack the structured method needed to transform raw qualitative inputs into coherent, actionable, and multi-layered design requirements. Many methods produce generalized suggestions that are not easily linked to real-world operational contexts, particularly in environments with high cognitive load, such as surgical paths.

To address these issues, this work introduces a user-centred interaction design framework that integrates user-centred design [6,7] with generative tools [8]. At its core lies the Persona-and-Scenario [9] method, which transforms raw user data into human-centred, narrative-based artifacts. By creating realistic personas and embedding them in contextual scenarios, the framework bridges the gap between abstract user feedback and concrete design requirements.

Starting from these assumptions, the main objectives and contributions of the paper are as follows:

(1) The development of a structured, scenario-driven interaction design framework specifically tailored to healthcare contexts.
(2) The integration of generative co-design techniques to uncover latent user needs; and
(3) The application of the framework through local and international co-design events conducted within the KEEPCARING project [10].

Accordingly, this study is guided by the following research questions:

- RQ1: How can generative and user-centred design methods be structured into a coherent framework for identifying requirements in healthcare platforms?
- RQ2: In what ways does the application of a Persona- and Scenario-based framework facilitate the identification of both explicit and latent user needs across diverse healthcare stakeholder groups?
- RQ3: How can insights from co-design activities be systematically converted into functional, usability, and interface requirements for healthcare systems?

The novelty of the proposed framework lies in:

- Innovative orientation because the proposed framework shifts from reactive evaluation to proactive envisioning of future workflows.
- Structured creativity because the application of the Persona-and-Scenario method bridges raw data to design requirements.
- Scalability because the framework is validated through local (in Italy) and international co-design events, ensuring adaptability.
- Holistic design because the framework addresses functional, usability, and interface requirements for multiple user roles (clinicians, managers, general users).
- Contextual depth because the framework embeds empathy and real-world scenarios into requirement inference, enhancing relevance and adoption.

Unlike conventional methods that focus on evaluating existing ideas, this framework empowers users to envision alternative futures, articulate hidden needs, and co-create solutions that directly address stress, burnout, and well-being challenges.

The proposed framework differs from conventional UCD approaches by not just refining existing ideas, but by utilizing the Persona-and-Scenario method [9] to enable users envision future interaction possibilities, anticipate stress-related challenges, and collaboratively develop innovative features. This forward-thinking perspective is particularly novel in the context of healthcare well-being platforms, where latent needs are often overlooked.

Additionally, the proposed framework outlines a cyclic process that converts user knowledge into structured functional, interface, and usability requirements.

Unlike other methods that generate descriptive insights without synthesis, this framework introduces a systematic translation mechanism that connects narrative-based user understanding to detailed system specifications. This represents a methodological advancement compared to existing UCD and co-design frameworks.

By combining personas, scenarios, co-design discussions, and structured requirement mapping, the framework effectively captures both explicit and latent user needs, providing a deeper contextual understanding that goes beyond traditional techniques.

Applied in the KEEPCARING project, the framework demonstrates how multidisciplinary stakeholders can collaboratively define services and functionalities for a web-based platform that supports resilience and professional health.

This framework offers an empathetic and contextually grounded approach that enhances usability, fosters innovation, and ensures that web-based platforms remain responsive to the evolving needs of health professionals. Accessibility and inclusive design evaluations ensure that healthcare applications can be used by individuals with disabilities, cognitive impairments, or limited digital and health literacy. Compliance with accessibility standards has become increasingly important in healthcare software development [11]. Inclusive UCD approaches also support broader health equity goals by addressing the needs of vulnerable and underserved populations [12].

Section 2 of this paper provides an overview of user-centred design methods for the collaborative design of platforms. This analysis allows the definition of the user-centred interaction design framework that is presented in Section 3. Following this, Section 4 describes the application of the user-centred interaction design framework in two co-design events: the Italian co-design event and the international co-design event. Finally, Section 5 provides a concluding discussion on the outcomes of the implementation of the proposed framework and future works.

## 2. Related Works

User-centred design (UCD) has become a foundational approach in healthcare technology development because it focuses on aligning digital solutions with the cognitive, emotional, and contextual needs of end users. These users often operate under intense time pressure and high cognitive load. Existing studies applying UCD in healthcare settings have leveraged a wide spectrum of qualitative, quantitative, and participatory methods to identify needs, validate usability, and refine workflows. For instance, retrieved studies focused on the development of eHealth platforms aimed at enhancing patient support [13], clinical information management tools designed to improve communication among healthcare professionals [14,15,16], and co-design models focused on enhancing user experience [2,5,17].

To better contextualize the proposed framework within existing UCD methods, studies that applied UCD in healthcare and in research addressing stress, burnout, clinician workflows, or well-being were selected and analysed.

Among UCD, focus groups [18] allow for in-depth discussions with small groups of users about the platform’s current state, new features, or concepts. This method helps to understand user attitudes, perceptions, and preferences in a group setting, but it often results in generalized feedback.

Methods like questionnaires and surveys [19] provide a straightforward way to gather user opinions on specific design elements, features, or functionalities. Users can be asked to rate or prioritize potential features, interface designs, or content structures. In addition, semi-structured and in-depth interviews are widely used to elicit user needs, expectations, and mental models. In healthcare design, interviews with clinicians and patients help capture domain knowledge, perceived barriers, and trust concerns [20]. While these methods allow for quick and easy collection of large amounts of data and user feedback, they provide raw data without the synthesis [21,22,23]. Interviews and observations provide in-depth, contextual insights into user workflows, but they have limited scalability. In contrast, surveys allow for statistical validation of user needs, though they offer less interpretive depth.

Participatory design and collaborative workshops [24,25,26,27] can also engage users actively in the design process. In healthcare applications, such methods have been shown to increase system acceptance, trust, and contextual relevance, particularly for patient-facing and mental health applications [28]. These methods can be structured around specific design challenges or broader platform goals, such as improving user experience or feature prioritization. They typically involve brainstorming, ideation, prototyping, and user testing. These methods provide benefits like immediate feedback, direct involvement, and a sense of ownership over the design process; they may generate ideas without structured user representation. These methods encourage collaboration and improvement, but they need considerable coordination and ethical oversight.

A comparative overview of the most widely used UCD methods is provided in Table 1. This table summarizes the strengths and limitations of focus groups, surveys, interviews, participatory workshops, and the Persona-and-Scenario method, highlighting their suitability for identifying user needs in healthcare contexts.

While interviews and observations yield a deep understanding, surveys and participatory design expand perspective and participation. The optimal strategy blends these approaches, guided by the healthcare context, user diversity, and project stage, ensuring that the resulting application truly enhances users’ needs.

When the purpose is to synthesize user data into actionable design tools, combining generative tools [6] with user-centred design allows individuals to uncover their latent needs and desires.

Generative tools go beyond evaluating existing ideas; these techniques encourage users to envision alternative futures and articulate hidden needs. These techniques enhance traditional user-centred design methods, which are often focused solely on engaging users in evaluating or refining existing ideas, products, or services. User-centred design methods that incorporate generative tools involve creative, forward-thinking activities that reveal users’ deeper needs and aspirations. These techniques enable users to explore challenges in application scenarios and express deeper insights about their experiences, obstacles, and requirements.

Among generative tools, the Persona-and-Scenario technique helps develop an understanding of participants’ experiences and challenges while also creating a vision for the future by envisioning an ideal state [29]. Other generative design tools include storytelling activities facilitated by illustrations and sketches [30], as well as creative prototyping exercises where participants create a physical representation of a concept or idea [31].

The Persona-and-Scenario method [32,33,34] is more effective than other user-centred design methods because it turns raw user data into engaging, human-centred, story-based artifacts. This method enables the collection of data that helps infer user requirements more clearly and realistically. By fostering engaging discussions characterized by empathy and simulating real-world interactions, the data gathered is more aligned with actual user needs.

This approach leads to better personalization and feature prioritization, and it provides a shared reference point for designers, developers, and decision-makers, ensuring that discussions about the platform remain user-focused and aligned with real-world scenarios.

It uses the Personas method that allows creating detailed fictional profiles based on real user data [9,26,35]. This method [34] is enhanced by incorporating a description of a specific situation in which the persona would use the platform. The scenario provides context for the story, illustrating how the persona interacts in that situation.

Despite the widespread use of the Persona-and-Scenario method in Human–Computer Interaction (HCI) and UCD, current approaches struggle to fully capture the complex, multi-layered dynamics, organizational interdependencies, and preventive well-being needs found in healthcare environments. Current methods mainly provide descriptive or evaluative insights that focus on individual user roles, lacking structured processes for converting generative knowledge into prioritized, testable design requirements. Additionally, empirical applications rarely incorporate indicators of physiological, contextual, and organizational stress, nor do they support collaboration across different roles during scenario development. Consequently, to our knowledge, no established framework effectively connects personas, future-state scenario envisioning, generative co-design, and requirement engineering into a rigorous, cyclical methodology that can promote proactive, resilience-oriented design in healthcare systems.

The proposed user-centred interaction design framework addresses this gap by advancing theory through a socio-technical, multi-role reinterpretation of personas; enhancing methodology with a structured five-phase process that links generative tools to requirement formalization; and contributing empirical knowledge through cross-stakeholder validation and the generation of multi-level, stress-informed design requirements. This innovative approach extends beyond traditional Persona-and-Scenario methods, directly addressing the complexity and urgency of promoting well-being, stress detection, and resilience among healthcare professionals.

The following sections will present the proposed framework based on the Persona-and-Scenario method.

## 3. The User-Centred Interaction Design Framework

This section outlines the co-design user-centred interaction design framework for acquiring knowledge on needs and requirements from various stakeholders (such as hospital-based doctors, nurses, medical students, nursing students, and hospital managers).

The provided user-centred interaction design framework has been defined to enhance the usability and accessibility of platforms [36,37], to facilitate the interaction process, and to prioritize the users’ needs and goals through their active involvement in the platform’s design.

For these purposes, the proposed framework is defined by combining user-centred design techniques with generative tools [6] by applying the Persona-and-Scenario method [32,33,34]. The framework follows a cyclical five-phase structure: (1) understanding users and their needs, (2) representing key user groups, (3) describing application scenarios, (4) collecting data in co-design events, and (5) converting insights into requirements. Each phase feeds into the next, enabling continuous refinement based on stakeholder feedback.

**Figure 1:**
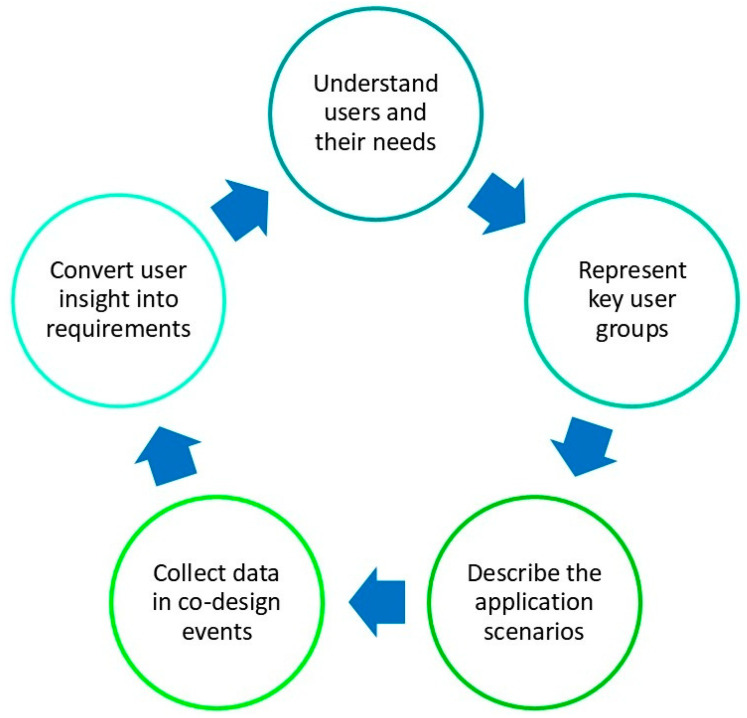
Phases of the user-centred interaction design framework.

The overall process through which user needs are elicited and transformed into requirements is summarized in Figure 1, which illustrates the five interconnected phases of the proposed framework. This visualization shows how iterative cycles support deeper insight generation and requirement refinement throughout the design process.

This framework aims at uncovering participants’ hidden needs and desires and creating new forms of interaction or functionalities that align with those needs and contexts by ensuring a user-centred approach throughout the process.

It provides a comprehensive view that integrates user behaviours, interface design, and feature prioritization in a cyclical way because it enables users to actively refine their extracted needs.

### 3.1. Understand Users and Their Needs

The analytical scope of the first phase is to define the stakeholder characteristics, focusing on understanding the users and their needs. This phase identifies key differences among user groups and gathers information about users’ roles, tasks, and context of use. It involves gaining a thorough understanding of user groups and the contextual conditions that will inform future design activities. This stage ensures that design decisions are based on empirical insights about users’ actual practices, needs, and environments, rather than on abstract assumptions.

The main goal of this phase is to define the design challenge and understand how the web platform will function. This involves identifying key user groups and shaping how users will interact with the platform. This phase lays the foundation for participatory and co-creative engagement by ensuring that all stakeholders have a shared understanding of the context and design objectives. A key task is identifying and recruiting relevant stakeholders, such as end-users, developers, and domain experts. Each of these participants brings tacit, experiential, and technical knowledge that collectively informs the design process. Engaging with them early on helps build trust and mutual understanding, which are essential for effective collaboration during co-design events and the ideation process.

### 3.2. Represent Key User Groups

The second phase provides the analytical synthesis of user characteristics through persona construction. The representation of key user groups ensures that the design of a web platform incorporates the diverse perspectives, needs, and experiences of its intended users. In the co-design process, users are active participants who contribute their experiential knowledge and contextual understanding throughout the design cycle. This participatory approach enhances the relevance and usability of the final system by grounding design decisions in real-world practices and values.

For this purpose, this phase aims at defining a user persona representing a fictional character designed to embody an ideal user. This includes their demographics, motivations, preferences, goals, and tasks. This phase synthesizes user data into relatable profiles that inform design decisions. While traditional co-design gathers explicit feedback from users, generative tools uncover deeper, often unspoken needs, and user personas transform these insights into fictional yet data-driven characters that represent key user types. These profiles ensure that the design process remains user-centred, allowing decisions to reflect real user goals, behaviours, and contexts, even when users are not directly involved in the later stages of development.

### 3.3. Describe the Application Scenarios

The third phase operationalizes personas within usage contexts through scenario development. The insights gathered from the previous phase on the user understanding and representation are transformed into situated narratives. These narratives illustrate how users might interact with the proposed web platform in realistic settings. This phase serves as a bridge between understanding the problem and developing concepts by converting abstract user needs into concrete, experience-based depictions of how the future system will be used. A user scenario is a narrative that illustrates how a user persona interacts with a specific feature within a particular context and situation. This phase focuses on envisioning and communicating possible use cases that demonstrate how the platform will support users’ goals, workflows, and interactions. Application scenarios serve as tools to align the design team and stakeholders around a shared understanding of the intended functionality, user experience, and contextual relevance. In co-design, the scenario is not created solely by designers; it is collaboratively developed with users and stakeholders, incorporating their lived experiences, expectations, and insights into everyday practices.

### 3.4. Collect Data in Co-Design Events

The fourth phase enables the systematic collection of data about the users. The data collection phase grounds the design in the actual needs, behaviours, and contexts of real users. This phase involves gathering insights collaboratively with stakeholders. It focuses on understanding users’ needs and goals, addressing current pain points with existing interaction systems, and considering the context of use along with stakeholders’ expectations and priorities. This information serves as the foundation for developing user-centred concepts and prototypes.

By utilizing the Persona-and-Scenarios approach, participants in the co-design event can empathize with the fictional character presented in the relevant application scenario. This approach allows them to actively discuss the challenges the character may face in that context and explore potential solutions to those issues. These co-design events facilitate discussions among users to identify shared needs and opinions by ensuring accuracy and shared understanding.

### 3.5. Convert User Insight into Requirements

The fifth phase translates the insights into prioritized design requirements. It focuses on converting qualitative insights, user needs, and contextual information collected during data gathering into specific, prioritized, and testable design requirements. These requirements act as a connection between research and design, ensuring that the final platform meets users’ actual needs, preferences, and constraints.

The aim is to transform raw data into structured insights that clearly articulate user behaviours, motivations, and challenges.

Transforming user insights into design requirements involves analysing data, identifying user needs, and converting these insights into specific, prioritized, and actionable design requirements. This phase is crucial to ensure that the design and development stages stay focused on the user and remain relevant to the context. The requirements identified in this phase are refined by repeatedly applying the framework.

## 4. The User-Centred Interaction Framework Implementation and Usage

To understand the effective application of the proposed framework, it was employed to capture the perspectives and needs of target users for the Change Management Platform (CMP) of the KEEPCARING project [5] for the purpose to increase awareness about stress and resilience, and to enable users to identify problems concerning stress and burnout.

### 4.1. Understanding Users and Their Needs in the KEEPCARING Project

In the primary phase of the framework, a clear criteria and procedures for participant identification and recruitment at both local (Italy) and international levels. This process encompassed identifying potential users of the KEEPCARING CMP, as well as ensuring that participants were fully informed on the co-design event’s purpose, structure, and outcomes while providing informed consent through appropriate documentation.

This phase identifies the different user groups that will utilize the CMP to achieve their goals. The aim is to engage individuals in diverse roles, including hospital-based doctors, nurses, medical students, nursing students, hospital managers, and professionals in medical informatics and health sector organizations. The recruitment process involved both direct and indirect communication strategies. (e.g., face-to-face meetings, emails, phone calls, social media). Efforts were made to ensure gender diversity in the participant pool, with a target of achieving a balanced gender representation of participants. All recruitment communications included a request for potential participants to register via the EU Survey platform [38].

All data collected during the co-design activities were anonymized and handled in compliance with relevant data protection regulations. This ensured that no personally identifiable information was retained during the analysis or reporting phases. Due to the sensitive nature of monitoring stress and burnout in healthcare, particular attention was paid to confidentiality, voluntary participation, and the non-evaluative use of shared information.

### 4.2. Represent Key User Groups in the KEEPCARING Project

In this phase, the Personas- and- Scenarios method was applied to identify user requirements for the web-based platform. Personas function as data-driven archetypes that represent different user groups, allowing the design team to create solutions that closely align with users’ goals, behaviours, and contexts of use.

To ensure that the CMP addresses the needs of diverse users, three personas were developed based on stakeholder input. Their profiles—summarized in Table 2, Table 3 and Table 4—represent healthcare professionals, hospital managers, and general end-users, respectively.

These personas are fictional characters developed within the Persona-and-Scenario method. They act as design artefacts that illustrate typical user roles, goals, and challenges relevant to the project context. Narrative details were included to support empathy and discussion during the co-design activities.

The persona in Table 2 reflects the needs, goals, and work-related challenges of nurses, doctors, and students engaged in high-demand clinical environments.

In Table 3, this persona characterizes the responsibilities, decision-making tasks, and well-being monitoring needs of healthcare managers overseeing clinical teams. The three representatives are provided as examples to guide discussions during the co-design events; therefore, the names used are pseudonyms.

Lastly, the persona in Table 4 represents individuals using the CMP primarily to access educational materials, training content, and self-regulation resources.

### 4.3. Describe the Application Scenarios in the KEEPCARING Project

The three personas described above were embedded into application scenarios used during the co-design sessions. Table 5 presents these scenarios, each depicting typical user interactions with the CMP. Each scenario describes a context in which one persona interacts with the CMP, illustrating use cases at the individual, organizational, and general levels.

The first scenario involves hospital-based doctors, nurses, and students using CMP to upload personal information, job details, and health data collected from wearable devices such as smartwatches and smart clothing. This scenario involves the first character described in the previous section (see Table 2) and pertains to a surgical professional seeking to alleviate stress stemming from their situation at the individual level. This scenario is categorized as the health professional level, as it focuses on the interaction process of professionals (who work in the surgical path) with the platform (see Table 4).

The second scenario focuses on healthcare decision-makers who interact with the CMP to visualize anonymized data regarding employees, such as work-related stressors and personal stress levels. In this scenario, the interaction process aims to provide insights into the distribution of stress types among employees and suggest interventions to reduce job stress, promoting a safe and resilient workplace. This scenario features the second character described in the previous section (refer to Table 3), and it focuses on effectively managing stressful situations while enhancing team well-being at the team level. It is categorized at the hospital manager’s level, as it describes the interaction process between the hospital manager and the platform (see Table 5).

Finally, the third scenario describes end-users seeking information about materials, publications, and project outcomes. This scenario features the third character outlined in the previous section (refer to Table 4) and is categorized at the general end-user level. It describes the interaction process between a general end-user and the platform (see Table 5).

This process enabled the conversion of experiential narratives, stakeholder reflections, and lived challenges into actionable specifications for the Change Management Platform (CMP). The progression from raw qualitative input to formalized requirements occurred through four interconnected interpretive stages: user understanding, scenario contextualization, collaborative sense-making, and requirement formalization. In the first stage, the creation of representative personas—such as Anna, Giulia, and Andrea—allowed the research team to synthesize diverse user characteristics, goals, and pain points into relatable profiles that encapsulated the behaviours and constraints of the platform’s intended users. These personas revealed underlying needs, including stress monitoring, timely alerts, minimal-effort interaction modalities, aggregated visualization of staff well-being, and intuitive access to support resources. For instance, the persona of Anna, a nurse balancing a high workload, emotional fatigue, and limited personal time, directly highlights the need for automated data capture, contextual information collection, and stress-related support mechanisms. Similarly, Giulia’s role as a hospital manager underscored managerial needs such as monitoring aggregated stress indicators and identifying organizational trends. The second stage involved embedding these personas into realistic scenarios that described how each user type would interact with the CMP. These scenarios provided concrete illustrations of workflows that required system support—for example, health professionals uploading contextual and physiological data, receiving personalized recommendations, or acting on alerts when risk thresholds were exceeded. Likewise, managers’ scenarios depicted the need for dashboards, reports, and suggestions for organizational interventions, while general users sought access to online training materials and stress-reduction content. Through these situated narratives, abstract user needs were translated into expected system behaviours, thereby bridging individual motivations with platform functionalities.

### 4.4. Data Collected in Local and International Co-Design Events

The three described scenarios were used to collaboratively discuss and explore user needs and requirements during the local and international KEEPCARING CMP co-design events. The local co-design event brought together hospital managers, hospital-based doctors, and experts in medical informatics to collaborate on the design of the services and functionalities of the KEEPCARING CMP. The main objective was to develop shared solutions based on the actual needs of users. The event took place on 24 February 2025, in a hybrid format (in person or online) to encourage broader participation among stakeholders, via the Teams platform [39]. A total of six stakeholders attended the event (three males and three females) and included three hospital-based doctors, one hospital manager, and two experts specializing in medical informatics and biomedical robotics. Moderators facilitated an equitable exchange of perspectives regarding user interactions, situational contexts, and preferred technological modalities for the CMP. The online tool SLIDO [40] was used to facilitate responses, allowing stakeholders to submit their answers online. Moderators guided discussions among the stakeholders to clarify their answers and to share their opinions, ideas, and suggestions for designing the services and functionalities of the KEEPCARING CMP.

The international co-design event involved various international stakeholders. It took place on 11 March 2025, and was conducted online via the Teams platform. A total of nine stakeholders attended, consisting of five males and four females. The participants were from both European and non-European countries. The attendees included: two hospital-based doctors, one hospital manager, one student, and five professionals with expertise in medical informatics and health sector organization. Following the same format as the national event, to collect responses, the SLIDO tool was utilized, allowing stakeholders to submit their answers online. The participants’ answers were displayed on the screen, and the moderators facilitated a discussion, encouraging stakeholders to elaborate on their responses, exchange viewpoints, and contribute ideas and suggestions for shaping the services and functionalities of the KEEPCARING CMP. To ensure that all participants could actively contribute to discussions during co-design events, a limited number of participants was involved. The primary goal in co-design is to cover key perspectives by focusing on achieving a saturation of ideas and including important stakeholder types.

**Figure 2:**
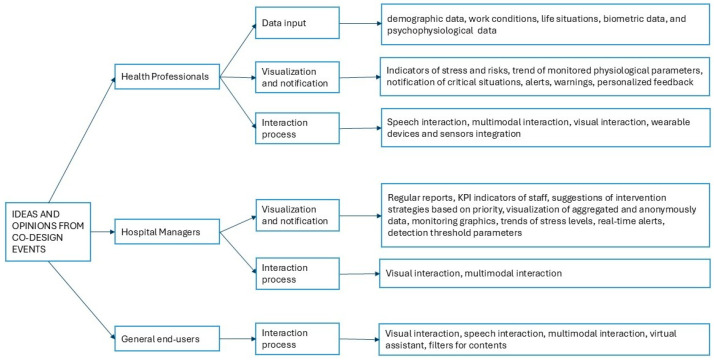
Data collected during co-design events.

From the two co-design events, ideas and opinions of different stakeholders for developing a digital platform to monitor stress and burnout were collected (see Figure 2). The questions proposed in the three application scenarios focused on gathering feedback on data to be collected for detecting stress and/or burnout among healthcare professionals, the preferred modalities for receiving notifications, and the ways of interacting with the platform.

To better understand stakeholder needs, the themes emerging from both co-design events were synthesized into three macro-areas, as illustrated in Figure 2. These macro-areas capture the breadth of expectations concerning data collection, interpretation, and user interaction with the CMP.

The qualitative data generated during the co-design sessions were analysed using a thematic analysis approach. Two researchers independently reviewed all collected inputs and conducted open coding to identify recurring concepts and patterns. These initial codes were iteratively compared, discussed, and refined, leading to the consolidation of themes into three macro-areas: (1) data input for stress and burnout detection and well-being improvement, (2) data visualization and notification of suggestions and alerts, and (3) interaction processes with the platform.

Figure 2 categorizes stakeholder insights into these three main macro-areas. These categories guided the extraction of functional, usability, and interface requirements.

Factors related to biometric data included biometric measurements collected through wearable devices. Finally, factors related to psychophysiological data contained perceived stress levels and the emotional states that could increase it, as well as the sleep quality that affects physical and mental health and work performance. Furthermore, stakeholders discussed the importance of anonymization for any data visible to managers and aggregation of sensitive data, as well as the need for encryption and password protection, informed consent, and the ability to refuse/disable features.

In the second macro-area, stakeholders discussed how and when health professionals and hospital managers desire to receive alerts and the kind of visualization and notifications. For example, for health professionals, the visualizations of indicators of stress and trends of physiological parameters are very important, and notifications and alerts are useful only when physiological or stress levels exceed risk thresholds or SOS functions for critical situations. Stakeholders also suggested the kind of support they would receive, including stress-reduction strategies and exercises, and personalized feedback based on individual stress levels. Data was also collected on the accessible channels indicated to deliver notifications (for example, smartphone alerts such as SMS or messaging apps, notifications via personal computer or smartphone notifications). From the hospital managers’ perspective, notifications are instead needed to identify organizational trends rather than individual issues. Visualization is then related to the need for regular weekly or monthly reports, key performance indicators related to staff well-being, and monitoring graphics for critical events or peaks in workload. Notifications are based on critical trends in staff stress levels and real-time alerts with suggestions for interventions and shift reorganization. Stakeholders also discussed the need to visualize aggregated and anonymous data, mainly related to absences, injuries, and shift change requests, that could indicate high levels of stress. The third macro-area collected data on the interaction process with the platform. From the health professionals’ perspective, stakeholders suggested interaction modalities that minimize cognitive and operational load during work activities, particularly in clinical environments like surgical units. For example, they suggested rapid interactions through speech and minimal manual input limited to quick actions such as pressing a button, visual interaction, and multimodal interaction. Automated data captured from wearable devices and sensors was considered particularly valuable. From the hospital managers’ perspective, stakeholders mainly discussed the need to have intuitive multimodal interaction and visual interaction through, for example, graphics and structured dashboards to support the decision-making processes. From a general end-user perspective, stakeholders suggested an interaction process with the platforms based on visual, speech, and multimodal interaction to provide a simple and intuitive experience for users seeking training and resources. Furthermore, stakeholders suggested personalized experiences aligned with the individual’s needs and interests, including a virtual assistant as well as filters for content that facilitate navigation.

### 4.5. Convert User Insight into Requirements in the KEEPCARING Project

The feedback from the co-design events was analyzed to provide valuable insights for designing a sustainable, user-friendly, and effective KEEPCARING CMP that reflects the specific needs and expectations of users.

**Figure 3:**
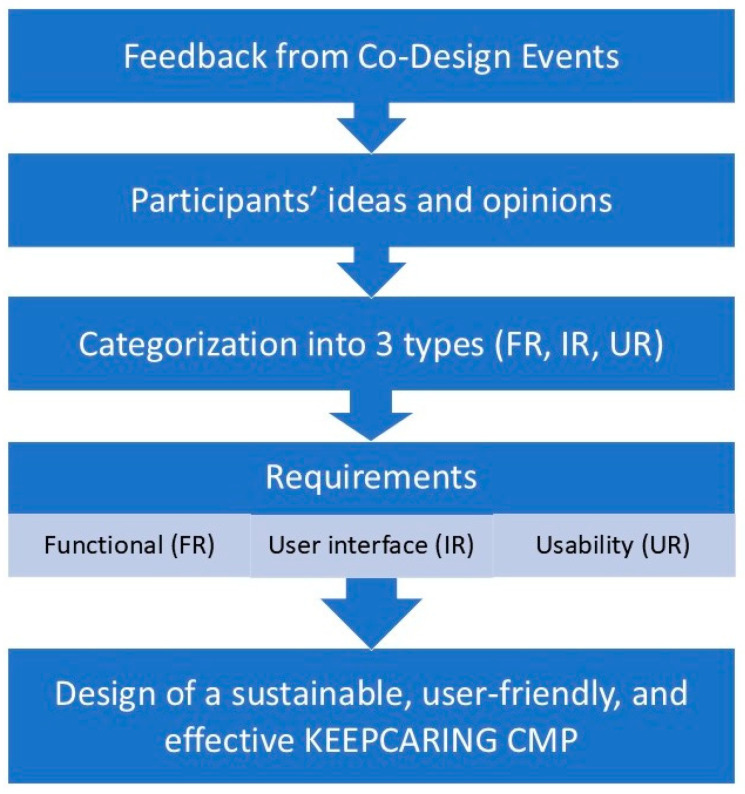
Workflow for gathering user requirements for the design of the KEEPCARING CMP.

This workflow shows how ideas from co-design activities were organized and classified into functional requirements (FRs), user interface requirements (IRs), and usability requirements (URs), forming a structured foundation for system design. Following data collection, stakeholder inputs were processed through the workflow illustrated in Figure 3, which maps raw insights to distinct requirement categories. This structured transformation supports systematic decision-making during platform development.

Insights from both co-design events were consolidated into the requirement categories summarized in Table 6, which include functional, interface, and usability requirements central to CMP design.

These requirements reflect the needs of health professionals, hospital managers, and general end-users and serve as the foundation for CMP development.

To further operationalize the requirement categories, Table 7 breaks down each requirement by user level, input sources, system outputs, and functional descriptions. This table operationalizes the requirements from Table 6 by mapping each to user groups and system behaviours.

The transformation of qualitative insights (described in Section 4.4 into structured user requirements followed a systematic and traceable reasoning process grounded in the Persona-and-Scenario method and validated through two co-design events. During the two co-design events, stakeholders collaboratively elaborated on these scenarios, contributing detailed insights related to data collection, visualization preferences, notification logic, and interaction modalities. This collaborative process enabled participants to refine and validate the needs emerging from personas and scenarios. Stakeholders emphasized the importance of multimodal interaction, limited cognitive load during clinical work, data anonymization, encryption, and the need for actionable alerts rather than continuous notifications. They further identified preferences for visual dashboards, threshold-based warnings, weekly or monthly aggregated reports, and personalized content suggestions aligned with users’ stress states. These discussions were systematically categorized into three thematic macro-areas—data input, visualization and alerts, and interaction processes—providing a structured foundation for the subsequent requirement elicitation phase. Finally, the synthesis of these qualitative insights resulted in a coherent set of functional, interface, and usability requirements, formally presented in Table 6 and Table 7. Each requirement traces back to specific qualitative evidence: monitoring and alerting functions (FR-04) emerged from clinicians’ stress and workload constraints; interoperability with wearable devices (FR-03) derived from scenarios involving physiological data collection; dashboard functionalities (FR-07) originated from managerial needs for aggregated organizational data; multimodal and low-effort interaction modalities (IR-03, IR-04) reflected both persona limitations and co-design discussions; and privacy and data protection measures (FR-06) were grounded in stakeholder concerns regarding sensitive health information. Usability considerations such as intuitiveness, accessibility, and multi-device compatibility (UR-01, UR-02, UR-03) were similarly shaped by general end-user expectations and the need to support individuals with varying levels of digital literacy. Through this structured and iterative process, qualitative input collected from diverse participants was systematically transformed into rigorous, evidence-based user requirements. This approach ensured that the CMP’s design remained strongly anchored in the lived realities, preferences, and operational constraints of its users, thereby enhancing its relevance, usability, and potential for successful adoption across healthcare contexts.

The ideas and opinions of participants underlined that the CMP should define three distinct registration levels: health professionals, hospital managers, and general end users. Each level is associated with specific data requirements, access permissions, and functional capabilities.

Users registered as health professionals are required to provide personal and work-related information during the registration process. In addition, the CMP collects physiological data from users’ wearable devices. These data are analysed to identify critical conditions associated with stress and burnout. Based on the analysis, the CMP generates alerts and delivers personalized recommendations aimed at mitigating these conditions. Such recommendations may include participation in self-regulation e-learning courses or engagement in virtual reality-based interventions designed to support deep relaxation and recovery.

For users registered at the hospital manager level, the CMP provides access to a dedicated dashboard that enables the monitoring of aggregated stress and well-being indicators among healthcare staff. This functionality supports the identification of critical stress and burnout situations at the organizational level and assists in the implementation of targeted strategies to improve employee well-being. When critical conditions are detected, the CMP proposes a set of intervention strategies and solutions, accompanied by predictive insights regarding their potential effectiveness for both healthcare professionals and managerial stakeholders.

At the general end-user level, the requirements primarily concern access to CMP content, the type of information provided, and the usability of the user interface. Consequently, accessibility and usability constitute key design considerations. The CMP should be intuitive and easy to use, particularly for individuals with limited familiarity with digital technologies. Content visualization should be clear and structured, employing icons and personalized views based on user preferences. Guided navigation should be supported through filtering mechanisms, virtual assistants, and context-aware recommendations.

To further enhance accessibility, the CMP should support flexible and multimodal interaction modalities. Multimodal interaction enables users to access platform content across diverse contexts, with voice-based interaction identified as a particularly practical and user-friendly option. The CMP should be designed to function as a personal well-being assistant rather than solely as a monitoring system. Accordingly, transparency and security in data management are essential to fostering user trust. Stakeholders emphasized the importance of proactive intervention mechanisms combined with personalized user experiences. User engagement should also be supported through visually appealing interface elements, including features inspired by game theory. However, the CMP is primarily intended for use outside working hours. During working hours—particularly in high-intensity environments such as surgical settings—interaction with the CMP should be minimal, efficient, and unobtrusive, relying on rapid input methods such as speech-based or multimodal interaction.

The requirements discussed reflect the emerging systemic needs in healthcare platforms. These needs address the increasing burden of stress and burnout among healthcare professionals, as well as the necessity for decision-support tools. They also emphasize the importance of protecting sensitive data and the urgent need to reduce the cognitive workload for clinicians.

Collecting contextual, emotional, lifestyle, and physiological data enables the early detection of burnout risk. This approach transforms the platform into a preventive health tool rather than merely a monitoring system. Additionally, combining self-reported data with data from wearable devices enhances the validity of the findings, lessens the burden on staff to manually input wellbeing information, and allows for interventions to occur before burnout leads to absenteeism or clinical errors.

These requirements focus on early detection and targeted interventions to address burnout proactively, rather than simply reacting to it. They provide tailored recommendations, virtual reality support, and adaptive learning pathways to help clinicians build resilience. In addition, the identified requirements reflect a holistic approach to supporting healthcare workers. They address the multidimensional nature of stress and burnout by combining personal monitoring, organizational analytics, and proactive interventions.

Overall, these requirements position the CMP as a comprehensive resilience support system. It aims to improve staff well-being, prevent burnout, and enhance team management.

## 5. Conclusions

This paper presented a user-centred interaction design framework to engage and guide users to actively design a digital platform aimed at monitoring the well-being, stress levels, and burnout of health professionals.

This framework has been designed to facilitate the collaborative design of a healthcare platform and to infer user requirements more clearly and realistically. The proposed user-centred interaction design framework was applied in two co-design events, one local in Italy and one international. During these events, different stakeholders, including hospital-based doctors, nurses, medical and nursing students, and hospital managers, were engaged to discuss services and functionalities to be included in the platform. This exchange of ideas and opinions yielded valuable insights into users’ expectations, needs, and challenges for a healthcare platform. Participants collaboratively identified data to be collected for detecting stress and/or burnout, preferred data visualization, notification of suggestions and alerts, and information on the interaction process. These insights were translated into user requirements and categorized into functional, usability, and user interface requirements to design a sustainable and user-friendly healthcare platform.

Although the proposed user-centred interaction design framework is applied in two co-design events, a formal evaluation of the framework will be conducted when a prototype of the CMP is implemented. The described work was designed to gather qualitative data, facilitate stakeholder engagement, and translate the insights into functional, interface, and usability requirements for the CMP. As such, the study focused on eliciting and structuring requirements rather than testing or validating the effectiveness of the framework. Despite this, the application of the framework provides valuable indications of its potential to strengthen requirement elicitation, stakeholder alignment, and design quality. First, the framework successfully captured a rich set of user needs and contextual behaviours by integrating personas, scenarios, and co-design discussions. These techniques revealed latent needs (e.g., multimodal interaction, threshold-based alerts, and anonymized data visualization) that are unlikely to emerge through interviews or surveys alone. This suggests that the framework enhances the breadth and depth of requirement elicitation.

Moreover, the use of collaboratively developed personas and scenarios facilitated a shared understanding among heterogeneous stakeholders, including hospital-based doctors, nurses, managers, students, and experts in medical informatics. Participants converged on common concerns such as privacy, workload reduction, low cognitive load, and the need for accessible visualization tools. The consistency of themes across the Italian and international co-design events indicates that the framework improved stakeholder alignment by providing a common language and reference point for discussing needs and functionalities. In addition, the resulting requirements documented in Table 6 and Table 7 reflect a high degree of specificity, contextual grounding, and testability. Requirements such as FR 04 (Parameter detection and alerts), FR 07 (Monitoring dashboard), and IR 04 (Ergonomic & multimodal design) show clear links to user narratives, work environments, and operational constraints identified during the co-design sessions. The structured mapping of user insights to requirement categories—functional, interface, and usability—suggests an improvement in design quality by ensuring that the requirements are not only user-centred but also actionable for developers.

In conclusion, while the framework has not yet undergone empirical evaluation, its application demonstrates promising indications that it enhances requirement elicitation, promotes cross-stakeholder alignment, and yields higher-quality, contextually relevant design requirements. As future work, a formal evaluation will be carried out to validate these preliminary conclusions and to determine the framework’s measurable impact on usability, adoption, and system effectiveness in clinical settings.

In addition, future work will investigate how the framework could be generalized and adapted to other healthcare contexts or extended to support additional well-being dimensions. Finally, AI-driven personalization, predictive analytics, and interoperability could be integrated with emerging health technologies to enhance proactive stress management and resilience-building.

## Figures and Tables

**Table 1:** Comparative table.

Method	Key Strengths	Limitations
Focus Groups [18]	Encourages idea generation; highlights shared needs and divergent opinions; efficient for multiple viewpoints.	Risk of dominant voices; confidentiality concerns in healthcare may inhibit honest feedback.
Surveys and Questionnaires [19]	Scalable; cost-effective; useful for validating trends identified qualitatively.	Limited depth; dependent on question design; low response rates.
Interviews [20]	Provides rich qualitative data; allows probing for context and emotion; builds empathy.	Time-consuming; small sample sizes; potential bias from the interviewer or user.
Participatory design and collaborative workshops [24,25,26,27]	Empowers users; fosters ownership and innovation; reveals deeper needs.	Requires skilled facilitation; not all users can articulate design ideas.
Personas-and-Scenarios [29]	Keeps focus on user types; supports empathy and design alignment.	Risk of oversimplification

**Table 2:** Representative profile of hospital-based clinicians and students.

Name: Anna Age: 46 Occupation: Nurse (Surgical Field) Work Schedule: 40 h/week, often with heavy shifts Family: Married, three children, and caring for her mother-in-law	
*Demographics:*	Age: Mid-40s Status: Married Children: 3 children Caregiver Role: Looking after her mother-in-law in addition to managing her own family
*Goals:*	Work–Life Balance: She strives to find balance between her demanding career and her personal life. Her goal is to maintain her health, well-being, and family commitments. Support from Colleagues: Anna thrives in environments where she feels supported by her team. She values collaborative teamwork and the reassurance that her colleagues are there to help when needed. Professional Fulfilment: Anna is motivated by helping her patients and feels a sense of purpose in her work, particularly in the surgical field, where the stakes are high.
*Challenges:*	Stress & Burnout: Anna faces constant stress due to the demanding nature of her work, often leading to emotional and physical exhaustion. Long shifts can take a toll on her energy levels, and she finds herself sacrificing sleep to meet the demands of her job and family. Time Constraints: With a busy professional life and caregiving duties, Anna struggles to find time for herself, often feeling as though there is not enough time in the day to manage everything effectively. Health Impact: The lack of sleep and high-stress levels are starting to impact Anna’s physical and mental health, causing fatigue and occasional anxiety. Limited Personal Time: She finds it difficult to engage in hobbies or activities that might help her relax due to the demands of her career and family responsibilities

**Table 3:** Representative profile of hospital managers.

Name: Giulia Age: 50 Occupation: Hospital Manager Workplace: Hospital (Surgical field) Team: Doctors, nurses, and administrative staff Work Schedule: Full-time, with high responsibility for staff coordination and hospital operations	
*Demographics:*	Age: 50 years old Status: Married Role: Hospital Manager Location: Urban healthcare centre
*Goals:*	Ensure Team Efficiency and Satisfaction: Giulia’s primary goal is to make sure her team works efficiently while maintaining good morale and well-being. Improve Staff Well-being: She wants to identify early signs of burnout, stress, or fatigue to intervene before issues escalate. To scheduling team workload while respecting staff needs and hospital demands. To make informed decisions regarding staff workload and wellness interventions.
*Challenges:*	Balancing staff availability, hospital needs, and individual preferences is time-consuming and prone to errors if done manually. She frequently observes fatigue and stress among healthcare staff, especially nurses working long shifts, and needs effective ways to monitor and prevent burnout. Without proper tools, it is difficult for Giulia to assess staff well-being or predict when interventions are necessary. Her role involves extensive reporting, coordination, and documentation, which can detract from time spent engaging with her team. She finds it difficult to engage in hobbies or activities that might help her relax due to the demands of her career and family responsibilities

**Table 4:** Representative profile of general end users.

Name: Andrea Age: 38 Occupation: an employee in a healthcare or high-stress professional field Work Schedule: Full-time, managing multiple responsibilities with frequent stress Location: Urban setting with access to web-based platforms	
*Demographics:*	Age: 38 years old Status: Married Role: Employee in the surgical field Location: Urban healthcare centre
*Goals:*	Reduce stress and manage emotional well-being effectively Improve self-regulation and resilience at work Enhance job satisfaction through better role crafting and skill development Use technology (online courses, VR tools) to learn new coping strategies Achieve a healthier work–life balance and regain motivation
*Challenges:*	High and persistent work-related stress Difficulty managing workload and emotional exhaustion Limited time to focus on self-care or skill development Unfamiliarity with new digital wellness tools Needs motivation and structure to maintain consistent use of online resources.

**Table 5:** Overview of application scenarios used in the co-design events.

Interaction Level	Scenario Description
*Health professional*	Anna is a 46-year-old nurse working in the surgical field. She works 40 h a week, often enduring heavy shifts and managing a high workload. Married with three children, she also takes care of her mother-in-law. After work, Anna frequently feels stressed, which leads to a lack of sleep. However, she feels supported by both her manager and her team.
*Hospital managers*	Giulia is a 50-year-old woman and a hospital manager responsible for overseeing a multidisciplinary team of doctors and nurses. She is in charge of scheduling the team’s shifts and requires tools to monitor their well-being. This will help her understand the necessary interventions to implement promptly.
*General end-user*	Andrea is feeling stressed and wants to access the platform’s online courses and virtual reality videos. These resources are designed to support self-regulation, improve job crafting and work skills, and reduce stress.

**Table 6:** Summary of functional (FR), interface (IR), and usability (UR) requirements identified during co-design events.

User Requirements	Description
*Functional (FR)*	FR-01 User registration and management FR-02 Contextual information collection FR-03 Wearable app interoperability and external system integration FR-04 Parameter detection and alerts FR-05 Stress-based support content FR-06 Privacy and data security FR-07 Monitoring dashboard FR-08 Critical situation advice FR-09 Mitigation strategies & solutions
*User Interface (IR)*	IR-01 Content visualization IR-02 Content navigation IR-03 Voice command activation IR-04 Ergonomic & multimodal design.
*Usability (UR)*	UR-01 intuitiveness UR-02 Accessibility UR-03 Multi-device compatibility

**Table 7:** Detailed specification of CMP user requirements by stakeholder level, input data, expected outputs, and description.

Requirement	Level	Input	Output	Description
FR-01 User registration and management	Health professionals, Hospital managers, and General end-users	User personal data	New user created in CMP	CMP allows user registration and profile management via a personal information card.
FR-02 Contextual Information Collection	Health professionals	User-provided contextual and physiological data	User stress classification	CMP collects detailed data (demographics, work, emotions, lifestyle, wearable data, etc.) to classify the user’s stress/burnout state.
FR-03 Wearable app interoperability and external system Integration	Health professionals	Wearable app connection	App synchronized with CMP	CMP must interoperate with dedicated apps for wearable devices.
FR-04 Parameter detection and alerts	Health professionals	Physiological and mood-related sensor data	Notifications, alerts, suggestions	CMP monitors parameters (sleep, HR, BP, VO2, stress indicators) and sends alerts and recommendations when thresholds are exceeded.
FR-05 Stress-based support content	Health professionals	User stress state data	Suggested support materials	CMP recommends KEEPCARING materials (e-course, VR relaxation, etc.) based on detected stress.
FR-06 Privacy and data security	Health professionals	Personal and professional data	Data protection mechanisms	CMP ensures anonymization, encryption, password protection, informed consent, feature acceptance options, and data deletion.
FR-07 Monitoring dashboard	Hospital managers	“View dashboard” command	Display of the dashboard	The CMP dashboard displays aggregated stress indicators, work hours, absenteeism, trends, and KPIs.
FR-08 Critical situation advice	Hospital managers	“View advice” command	Display of prioritized advice	CMP shows analysis of critical issues, threshold alerts, email summaries, and weekly reports with priority levels.
FR-09 Mitigation strategies & solutions	Hospital managers	“Process solutions” command	Suggested strategies	CMP suggests solutions to mitigate stress.
IR-01 Content visualization	Health professionals, Hospital managers, and General end-users	Visualization commands	Selected visualization mode	CMP supports icons, tree lists, AI/ML personalized views, and emoji-based content visual alignment.
IR-02 Content navigation	Health professionals, Hospital managers, and General end-users	Navigation commands	Activated navigation mode	CMP navigation through filters, user-based suggestions, a virtual assistant, and virtual reality.
IR-03 Voice command activation	Health professionals, Hospital managers, and General end-users	Voice commands	Activated command and language customization	CMP supports voice commands and automatic adaptation to the user’s language.
IR-04 Ergonomic & multimodal design	Health professionals, Hospital managers, and General end-users	Ergonomic design principles	Multimodal interface	CMP interface supports touch, speech, and visual interaction with ergonomic design.
UR-01 Intuitiveness	Health professionals, Hospital managers, and General end-users	Usability design guidelines	Tasks should require minimal cognitive effort (important for clinical settings such as surgical units). Icons and navigation elements must be self-explanatory.	The CMP interface must enable users—especially health professionals with limited time—to quickly understand how to access key functions without training.
UR-02 Accessibility	Health professionals, Hospital managers, and General end-users	Usability design guidelines	Text size, contrast, and layout must be adjustable or adequately legible. Interaction must be inclusive across different user types (professionals, managers, general users).	The CMP interface must comply with accessibility principles relevant for diverse users, including those with limited digital literacy or temporary stress-induced cognitive overload.
UR-03 Multi-device compatibility	Health professionals, Hospital managers, and General end-users	Usability design guidelines	Key functionalities (e.g., alerts, dashboards, and content access) must remain usable across devices. Layouts must adapt responsively to screen sizes.	The CMP must function seamlessly across different devices.

## Data Availability

The datasets presented in this article are not available because there are privacy restrictions according to the informed consent signed by the participants, and they are part of the ongoing activities of the project.
